# Depression in the elderly: Does family system play a role? A cross-sectional study

**DOI:** 10.1186/1471-244X-7-57

**Published:** 2007-10-25

**Authors:** Ather M Taqui, Ahmed Itrat, Waris Qidwai, Zeeshan Qadri

**Affiliations:** 1 Medical College. The Aga Khan University, Karachi, Pakistan; 2 Department of Family Medicine. The Aga Khan University, Karachi, Pakistan; 3 Department of Community Health Sciences. The Aga Khan University, Karachi, Pakistan

## Abstract

**Background:**

The most common geriatric psychiatric disorder is depression. The role of family systems in depression among the elderly has not been studied extensively. It has been suggested that urbanization promotes nucleation of family systems and a decrease in care and support for the elderly. We conducted this study in Karachi, a large urban city of Pakistan, to determine the relationship between the type of family system and depression. We also determined the prevalence of depression in the elderly, as well as correlation of depression with other important socio-demographic variables.

**Methods:**

A cross-sectional study was carried out in the premises of a tertiary care hospital in Karachi, Pakistan. Questionnaire based interviews were conducted among the elderly people visiting the hospital. Depression was assessed using the 15-item Geriatric Depression Scale.

**Results:**

Four hundred subjects aged 65 and above were interviewed. The age of majority of the subjects ranged from 65 to 74 years. Seventy eight percent of the subjects were male. The prevalence of depression was found to be 19.8%. Multiple logistic regression analysis revealed that the following were significant (*p < 0.05*) independent predictors of depression: nuclear family system, female sex, being single or divorced/widowed, unemployment and having a low level of education. The elderly living in a nuclear family system were 4.3 times more likely to suffer from depression than those living in a joint family system (AOR = 4.3 [95% CI = 2.4–7.6]).

**Conclusion:**

The present study found that residing in a nuclear family system is a strong independent predictor of depression in the elderly. The prevalence of depression in the elderly population in our study was moderately high and a cause of concern. The transition in family systems towards nucleation may have a major deleterious effect on the physical and mental health of the elderly.

## Background

Depression is recognized as a serious public health concern in developing countries. The Global Burden of Disease study showed that depression will be the single leading cause of Disability Adjusted Life Years by 2020 in the developing world [1].

Depression is the most common psychiatric disorder among the elderly which can manifest as major depression or as minor depression characterized by a collection of depressive symptoms [2]. Many studies have indicated severe under-recognition and under-treatment of depression in the elderly, even in developed counties [3-5].

Between the years 2000 and 2050, the world wide proportion of persons over 65 years of age is expected to more than double, from the current 6.9% to 16.4% [6]. As health care facilities improve in countries, the proportion of the elderly in the population and the life expectancy after birth increase accordingly. This is the trend which has been seen in both developed and developing countries [7]. It is commonly believed that the majority of the elderly population resides in developed countries. However, this is a myth, as about 60% of the 580 million older people in the world live in developing countries, and by 2020, this value will increase to 70% of the total older population [8].

Pakistan is a developing country with poor health indicators. Currently it is the sixth most populous country in the world with an estimated population of 166 million [9]. The average life expectancy after birth is 62. The elderly (65+ years) form 4% (6.6 million) of the total population [9]. The elderly in Pakistan face a large number of psychological, social and physical health problems and the majority is dissatisfied with the available health care services [10].

A high level of prevalence of depression in community samples has been noted by most of the studies done in Pakistan. A systematic review by Mirza and Jenkins revealed that the overall mean prevalence of anxiety and depressive disorders in the community was 34% [11] with the highest level reported to be 66% [12].

In Pakistan, the joint family system has been the prevalent family system [13]. However, in recent times, urbanization has led to alterations in existing family structures in the country, especially the larger cities. It has been suggested that urbanization leads to households becoming less extended and more nuclear and that this trend would be observed in developing countries [14]. Hence, Karachi being the largest city of Pakistan [9], has witnessed the greatest impact of urbanization, and is a good candidate to study the corresponding changes in family systems.

In Pakistan, the majority of the elderly depend on their children and/or grandchildren for support: physical, social as well as financial [15]. The provision of this support, especially physical support is more practical in a joint family system. Mason has suggested that urbanization is likely to erode the family's ability to care for elderly members as well as decrease co-residence of adult children with the elderly [13]. Therefore, we hypothesized that the elderly residing in a nuclear family system would be at a higher risk of suffering from depression than those residing in a joint family system. We believe this risk factor holds much more importance in our society than western societies and its influence must be studied. We conducted this study to determine the association between depression in the elderly and family systems. We also determined the prevalence of depression in the elderly, as well as correlation of depression with other important socio-demographic variables.

## Methods

### Study design and study site

This cross-sectional study was conducted among elderly subjects visiting the Aga Khan University and Hospital (AKUH); a private, tertiary care, teaching hospital in Karachi, Pakistan.

Karachi is the largest city of Pakistan and holds about 10% of its population [16]. AKUH is situated in the heart of the city and the large majority of patients come from all over Karachi to avail the health facilities. AKUH has 542 beds in operation and provides services to over 38,000 hospitalized patients and to over 500,000 outpatients annually. As opposed to government hospitals in Karachi, AKUH has a much higher standard of care and it receives a larger proportion of high socioeconomic class patients. However, AKUH also has clinics in a Community Health Centre (CHC) where the consultation fee is low. The CHC has family medicine clinics. In addition, it also provides specialist services. It caters to patients from all socioeconomic classes. The waiting area of the CHC clinics was chosen as the study site because the majority of people there are from a low or medium socioeconomic class, which is more representative of the population of Karachi.

### Study sample and data collection

All consenting subjects, aged 65 years and above, and who were permanent residents of Karachi (residing more than 2 years in the city) were included in the study. The subjects comprised of both patients and attendants. Exclusion criteria included subjects who could not understand Urdu and those who were unable to complete the interview. The time period for the data collection was one month.

We required a sample size of 426 to fulfill our objectives at a 95% confidence level. This sample size was calculated assuming a 50% prevalence of depression, 5% bound-on error and 10% non-response rate. We assumed the prevalence of depression as 50% as no local study was found which assessed the prevalence of depression in an elderly population. The studies from regional countries like India were also not considered because there was a large variation in reported depression prevalence rates: 6% to 50% [17,18].

A probability sample was obtained by approaching all the subjects in a consecutive manner. The interviews were conducted by senior medical students. The interviews were questionnaire based and were conducted in the waiting areas of the CHC clinics. Written informed consent was obtained. The objectives of the study and the right to withdraw at any time were explained. Strict confidentiality was ensured. The study was conducted in compliance with 'Ethical principles for medical research involving human subjects' of Helsinki Declaration. The study protocol was discussed with supervising faculty for possible ethical concerns.

### Questionnaire

The questionnaire was divided into two parts. The first part comprised of socio-demographic information. The second part comprised of a scale for measuring depression in the elderly.

After completion of the English version of the questionnaire, an Urdu version was developed, as we did not expect the majority of the elderly subjects to be well versed in English. Three independent translations to Urdu and back-translations were done and the best worded script was selected [See additional files 1, 2 and 3].

Pre-testing was carried out on twenty-five elderly subjects to screen for potential problems in the questionnaire. No significant changes were made in the questionnaire. The results of the pre-test were discarded. The interviewers discussed the questionnaire thoroughly before data collection, to decrease interviewer bias and variability.

#### Part 1

Socio-demographic information covered a diverse set of parameters namely; age, sex, marital status, education, living conditions, caregivers, employment status, financial support and the type of family system the subject was currently residing in.

#### Part 2

The 15-item Geriatric Depression Scale (GDS) was incorporated in our questionnaire and was read out in the interviews. Each interviewer underwent training in interviewing technique for GDS by a psychiatrist.

Despite the availability of five validated questionnaires for assessing depression in Urdu speaking populations [19], we chose the GDS because the presentation of depression in the elderly is different, with less emphasis on somatic indicators and more on affective symptoms [20,21]. Hence, the GDS is better suited in identifying depression in the elderly [22]. When applied as a screening tool on cognitively intact elderly people, it has proven to be on par or better than other self-rating depression scales. Several studies have reported good correlations between the long (30-item) and short (15-item) form of the GDS [23-25]. The cut-off used for detecting depression was a score of 5 or above on the GDS. Using this cut-off, a high sensitivity and specificity of the 15-item GDS has been reported [26,27]. This scale has been used both as a self-administered questionnaire and read out in interviews [22].

### Definitions

Joint Family System (JFS) comprises of two or more nuclear families that form a corporate economic unit [28].

Nuclear Family System (NFS) is a family unit consisting of parents and their dependent children [29]. A family unit consisting of a single married or unmarried individual was also grouped under a nuclear family system.

### Statistical Analysis

The data was entered in Epi Data version 3.1 and analyzed in Statistical Package for Social Sciences 13.0 (SPSS, Inc., Chicago, IL, USA). Descriptive statistics were performed. The results were recorded as frequencies, means ± standard deviations (SD) and p-values. Tables and figures were used for comprehensive viewing of the results.

Univariate comparison of variables was done between the depressed and non-depressed group. The Chi-square test and Fisher's exact test were used for categorical variables. The variables which were biologically meaningful or had a p-value < 0.25 on univariate analysis were subjected to a stepwise multiple logistic regression analysis to determine which factors were independent predictors of depression in the elderly subjects [30]. The Hosmer and Lemeshow goodness-of-fit test was also used to determine the final model [30]. Unadjusted as well as adjusted odds ratios were recorded with a 95% confidence interval for each. Unless stated otherwise, a p-value of < 0.05 was taken as the criteria of significance for all purposes.

## Results

Four hundred and seventy two potential subjects were approached in a consecutive manner with the request for participation in the study. Four hundred and sixteen of the subjects agreed to participate, giving a response rate of 88.1%. The majority (80.4%) of the non-responders were females. Sixteen subjects did not complete the interview due to lack of time. In the end, 400 subjects were included in the analysis.

Our sample comprised of a total of 400 elderly people visiting the Aga Khan University and Hospital (AKUH). The majority (86.8%) of the subjects fell in the age range of 65–74 years. The mean age of the subjects was 69 years. Of all the subjects, 78% were male, 21.2% were unmarried (single, divorced, widowed or separated) at the time of study and 63.3% were educated. A large proportion (59.9%) of males were unemployed or retired. Four percent of the subjects were living alone and 11.8% had no caregivers present. Out of the 400 subjects, 19.5% of the subjects screened positive for depression on the GDS.

Table 1 shows the socio-demographic variables and compares these variables between joint and nuclear family systems. The subjects living in the joint family system (56.5%) outnumbered those in the nuclear family system (43.5%) by a small margin. There were significant differences in the distribution of marital status (p = 0.013) and education (p = 0.01) between the two family systems.

**Table 1:** Sociodemographic profile of subjects

**VARIABLES**	**TOTAL (n = 400)**	**Joint Family System (n = 226)**	**Nuclear Family System (n = 174)**	p-value*
	**n (%)**	**n (%)**	**n (%)**	
		
**Age**				
65–74	347 (86.8)	190 (84.1)	157 (90.2)	
75–84	43 (10.8)	28 (12.4)	15 (8.6)	
85+	10 (2.5)	8 (3.5)	2 (1.1)	0.137
Mean ± S.D	69 ± 5.1	69.4 ± 5.6	68.4 ± 4.4	

**Gender**				
Males	312 (78)	170 (75.2)	142 (81.6)	
Females	88 (22)	56 (24.8)	32 (18.4)	0.126

**Marital Status**				
Single	11 (2.7)	3 (1.3)	8 (4.6)	
Married	315 (78.8)	172 (76.1)	143 (82.2)	
Widowed/Widower	69 (17.3)	49 (21.7)	20 (11.5)	
Divorced/Separated	5 (1.2)	2 (0.9)	3 (1.7)	0.013

**Education**				
Illiterate	51 (12.8)	38 (16.8)	13 (7.5)	
Can Read/write	32 (8)	17 (7.5)	15 (8.6)	
Primary	64 (16)	40 (17.7)	24 (13.8)	
Secondary	100 (25)	60 (26.5)	40 (23.0)	
Inter	48 (12)	24 (10.6)	24 (13.8)	
Grad/Post-grad	105 (26.2)	47 (20.8)	58 (33.3)	0.010

**Living Conditions^†^**				
Living alone	16 (4)	0 (0)	16 (19.2)	
Living with spouse	303 (77.9)	164 (73.5)	139 (83.7)	--^‡^
Living with children	292 (75.1)	216 (96.9)	76 (45.8)	

**Caregiver(s)**				
Present	353 (88.2)	205 (90.7)	148 (85.1)	0.082
Absent	47 (11.8)	21 (9.3)	26 (14.9)	

**Employment Status (Males)^§^**				
Employed	125 (40.1)	69 (40.6)	56 (39.5)	0.836
Retired/Unemployed	187 (59.9)	101 (59.4)	86 (60.5)	

**Financial Support**				
Other sources ^¶^	302 (75.5)	183 (81)	119 (68.4)	
Self only	98 (24.5)	43 (19)	55 (31.6)	0.004

**Depression**				
Present	78 (19.5)	27 (11.9)	51 (29.3)	<0.001
Absent	322 (80.5)	199 (88.1)	123 (70.7)	

* The Chi-square test and Fisher's exact test was used to calculate the p-value

^† ^This parameter will have multiple responses and the total of each column will not add up to 100%

^‡ ^p-value is not applicable here because this parameter has multiple responses and each category is not mutually exclusive.

^§ ^For this parameter, only males are being taken into account. Therefore n = 312 for the TOTAL column, n = 170 for the JFS column, and n = 142 for the NFS column.

^¶ ^Other sources include children, pension, other family members and charity.

**Figure 1:**
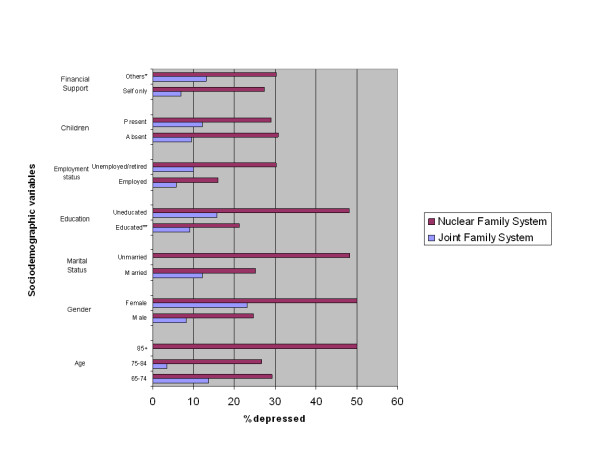
**Comparison of socio-demographic variables among depressed subjects in the two family systems**. *Other sources include children, pension, other family members and charity. **Educated = Having passed more than eight grades.

Figure 1 compares the two family systems for percentage of depressed subjects with selected socio-demographic variables. For each socio-demographic variable, the percentage of depressed subjects is higher in the nuclear family system.

Table 2 shows the univariate comparison of socio-demographic variables between the depressed and non-depressed group. In the univariate analysis, females were found to be 2.6 times more likely to suffer from depression compared to males (p < 0.001). Married people were less likely to suffer from depression compared to those who did not marry or who were separated or widowed (p < 0.001). The subjects living in a nuclear family system were more likely to suffer from depression than those living in a joint family system. Other factors that showed a significant association with depression in the subjects included being uneducated (p = 0.03), living alone (p < 0.001), being childless (p < 0.001), and being unemployed (p < 0.001).

**Table 2:** Univariate comparison of variables among depressed and non-depressed subjects

VARIABLES	DEPRESSED n (%)		UNADJUSTED OR* (95% CI**)
		
	**Yes**	**No**	
**Age (p = 0.270)**			
65–74 years	72 (92.3)	275 (85.4)	1
75–84 years	5 (6.4)	38 (11.8)	2.4 (0.3–18.9)
85+ years	1 (1.3)	9 (2.8)	1.2 (0.1–11.4)

**Gender (p < 0.001)**			
Male	49 (62.8)	263 (81.7)	1
Female	29 (37.2)	59 (18.3)	2.6 (1.5–4.5)

**Marital Status (p < 0.001)**			
Married	49 (62.8)	266 (82.6)	1
Single^†^	5 (6.4)	6 (1.9)	4.5 (1.3–15.4)
Divorced/Widowed/Separated	24 (30.6)	50 (15.5)	2.6 (1.5–4.6)

**Education (p = 0.003)**			
Educated^‡^	38 (48.7)	215 (66.8)	1
Uneducated	40 (51.3)	107 (33.2)	2.1 (1.3–3.5)

**Living Conditions (p < 0.001)**			
Living with others	69 (88.5)	315 (97.8)	1
Living alone	9 (11.5)	7 (2.2)	5.9 (2.1–16.3)

**Caregiver(s) (p = 0.743)**			
Present (353)	68 (87.2)	285 (88.5)	1
Absent (47)	10 (12.8)	37 (11.5)	1.1 (0.5–2.4)

**Family System (p < 0.001)**			
Joint Family System (226)	27 (34.6)	199 (61.8)	1
Nuclear Family System (174)	51 (65.4)	123 (38.2)	3.1 (1.8–5.1)

**Children (p < 0.001)**			
Yes	64 (82.1)	305 (94.7)	1
No	14 (17.9)	17 (5.3)	3.9 (1.8–8.4)

**Frequency of Children visits (n = 77) (p = 0.496)**			
At least once a month	4 (18.2)	14 (25.5)	1
Less than once a month	18 (81.8)	41 (74.5)	1.5 (0.4–5.3)

**Employment Status (p < 0.001)**			
Employed	13 (16.7)	112 (34.8)	1
Unemployed/Retired	36 (46.1)	151 (46.9)	2.1 (1.1–4)
Housewives	29 (37.2)	59 (18.3)	4.2 (2–8.7)

**Financial Support (p = 0.75)**			
Other sources^§^	60 (76.9)	242 (75.2)	1
Self only	18 (23.1)	80 (24.8)	0.9 (0.5–1.6)

* OR = Odds Ratio

** CI = Confidence Interval

^† ^These were the respondents who never married.

^‡ ^Educated = Having passed more than eight grades.

^§ ^Other sources include children, pension, other family members and charity.

The following variables were subjected to the multiple regression analysis: 'age', 'gender', 'marital status', 'education', 'living conditions', 'family system'. Table 3 shows the independent predictors of depression in the subjects. Multiple logistic regression analysis revealed the following factors to be independent predictors of depression in our sample: Nuclear family system, female gender, being unemployed or retired, being uneducated, being single and being divorced/widowed/separated. The subjects in a nuclear family system were 4.3 times more likely to suffer from depression than subjects in a joint family system (AOR = 4.3 [95% CI = 2.4–7.6]).

**Table 3:** Multiple logistic regression analysis showing independent predictors of depression in the subjects

**VARIABLES**	**DEPRESSED n (%)**	ADJUSTED OR*	95% CI**
**Family System (p < 0.001)**			
Joint Family System	27 (11.9)	1	
Nuclear Family System	51 (29.3)	4.3	2.4–7.6

**Gender (p = 0.003)**			
Male	49 (15.7)	1	
Female	29 (33)	3.5	1.5–8.1

**Marital Status (p = 0.023)**			
Married	49 (15.6)	1	
Single^†^	5 (45.5)	4.1	1.1–15.3
Divorced/Widowed/Separated	24 (32.4)	2.0	1.0–4.0

**Employment Status (p = 0.035)**			
Employed	13 (10.4)	1	
Unemployed/Retired	36 (19.3)	2.2	1.1–4.4

**Education (p = 0.003)**			
Educated^‡^	38 (15)	1	
Uneducated	40 (27.2)	2.3	1.3–4.1

* OR = Odds Ratio

** CI = Confidence Interval

^† ^These were the respondents who never married

^‡ ^Educated = Having passed more than eight grades.

## Discussion

The present study revealed that the elderly living in a nuclear family system were more likely to be depressed as compared to those in a joint family system. The prevalence of depression was moderately high among the elderly in our study population. About one in five of the elderly being depressed is a cause of concern. It was also shown that several important socio-demographic variables had a significant association with depression in the elderly.

### Prevalence of Depression

The prevalence of depression in the elderly in our study was 19.5%. No published data was found which reported the prevalence of depression in the elderly in Pakistan. A recent systematic review revealed that the prevalence of anxiety and depressive disorders in Pakistan was 34% [11]. Local studies have shown great variation in the prevalence of depression: 10% [31] to 66% [12]. It is unclear whether this variation reflects methodological differences in study designs and instruments or true differences in prevalence. The prevalence among the elderly in our study is high and a cause of concern. It cannot be said whether the prevalence is higher or lower in the elderly as compared to adults (15–64 years), because there are no large scale epidemiological studies present.

The prevalence of depression in Caucasian elderly populations in the West varies from 1% to 42% [32]. As for developing countries, there is paucity of literature on elderly populations. India is a neighboring country of Pakistan with a similar socio-demographic structure. The prevalence rates for depression in community samples of elderly in India have varied from 6% to 50% [17,18]. The prevalence computed in our study lies in the lower part of this spectrum.

### Socio-demographic Variables

The socio-demographic variables which were significantly associated with depression in the multiple logistic regression analysis are discussed in detail here. Each variable is also discussed in the context of family systems as well.

### Family Systems

The traditional family system in South Asia is the joint family system [13]. The greater proportion of the population in Pakistan (66%) lives in rural areas where the main occupation is agriculture [9]. The joint family system is predominant in the rural areas. One of the main advantages of a joint family system is the availability of a large workforce for occupations which demand one, like agriculture. Also, housing costs are shared. There is usually a joint economic production from the male members of the household.

In a joint family system, there exists a strong differentiation of authority across generations, and a relatively passive role of females. The elderly male holds an authoritative place in the family because he controls the family's property and exerts control over the younger generation. Their position demands obedience and loyalty. On the contrary, elderly females possess limited authority over the household and its matters. Despite the disparity in roles, both elderly males and females are shown respect by the younger generation in the family.

In urban areas, dissolution of the joint family system is likely due to the problems of housing (large families being unable to live under one roof) and occupational alternatives which result in the younger generation undergoing separation of economic production. One of the main consequences of this separation is a loss of the 'elderly power' over the younger generation [33,34]. This promotes nucleation of family systems, since the elderly male has lost his source of power over the younger generation: the possession of the family's resources. Nucleation leads to a decrease in co-residence of the elderly with adult children and therefore a decrease in care and support of the elderly.

Mason and Bongaarts have both suggested that urbanization would lead to nucleation of family systems in developing countries and a decrease in the support of the elderly [13,14]. A companion paper has suggested that more and more people were adopting the nuclear family system in Karachi [15]. This suggests a possible erosion of the joint family system in Karachi.

Consistent with the effects of urbanization on elderly care and family structures discussed above, the current study found that the elderly living in a nuclear family system were four times more likely to suffer from depression than those living in a joint family system. A joint family is much better able to provide support to the elderly than a nuclear family system, especially physical, social and emotional support. Financial support of the elderly can still be ensured by children or relatives even if they reside elsewhere. A study done in India showed that living in a joint family system was associated with a favorable outcome in elderly suffering from depression [35].

When the elderly contract a chronic somatic illness and undergo functional decline, it makes them strongly susceptible to depression [32,36]. In a nuclear family system, there are less caregivers to provide physical support and this can contribute to depression because the morbidities associated with chronic illnesses can be compounded if there is insufficient physical support. In a joint family system, the elderly have more interactions with people at home. These social interactions are experienced less in a nuclear family system. Prince et al. showed that social support deficits had a strong association with depression in the elderly [37]. Nuclear family systems also lack the emotional support which is provided to the elderly in a joint family system. Multiple other problems like spousal death, accidents, ill-health, or financial burdens are much more bearable by the elderly if family support is present.

Four studies done in Pakistan evaluated the association between depression and family systems [12,31,38,39]. Three of these studies were done by the same author using the same instrument in rural areas of Pakistan. Out of the four studies, two reported no association with family systems [38,39], one reported that women living in nuclear family systems were more likely to suffer from depression [12] and one reported that females living in joint family systems were more likely to suffer from depression [31]. These findings were not conclusive on the subject. Our findings indicate that living in a nuclear family system is a stronger risk factor for depression in the elderly than adults.

### Gender

The prevalence of depression in female elderly subjects was twice that in males (33% vs. 15.7%). Female gender was also a significant risk factor for depression on multivariate analysis. This finding is concurrent with the contemporary literature. Two recent reviews showed that female gender is consistently a significant risk factor for depression in the elderly [32,40]. Most of the studies done in Pakistan also report a high prevalence of depression in women, ranging from 25% to 66% [12,31,39,41]. Female gender has been significantly associated with depression in most of the studies including one which showed a significant association between depression and females who lived in a nuclear family system [12,41,42].

When comparing gender in Pakistan, female gender has been consistently associated with a higher prevalence of depression. It is likely that this high prevalence of depression in adult females (15–64 years) has carried over into old age. Mason has suggested that the problem of care for elderly females in countries like Pakistan is likely to be acute [13]. Elderly females are more likely to be depressed than elderly males because the extensive gender and generational asymmetries in a joint family system are likely to put elderly females at a particular risk of non-support, especially in the face of changes that degrade the family's traditional system of care [13]. Death of the spouse could lead to dissolution of the joint family system, leaving the elderly female without support.

### Education

In our study, a low level of education was directly associated with depression in the elderly subjects. Many studies have reported this finding [36,43], including studies in developing countries [44,45]. A large number of studies in Pakistan have also reported a significant association of low educational status with depression [12,31,39,41].

A low level of education makes it difficult for an individual to accomplish certain tasks satisfactorily in an urban city like Karachi. Examples include consultation with doctors, filling out forms in English and managing household finances. The elderly who face such problems are at a greater risk of suffering from depression.

The problem may be compounded if the elderly are residing in a nuclear family system. The elderly with a low level of education are generally dependent on family members and relatives for the above mentioned chores. If there are no family members to take care of these issues, as in a nuclear family system, it could contribute to depression in the elderly. This is illustrated in Figure 1, which shows that while uneducated subjects as a whole are more depressed, those uneducated people who are living in a nuclear family system are more depressed than subjects residing in a joint family system.

### Marital Status

The prevalence of depression was found to be significantly higher in the elderly who were single (never married), widowed, divorced or separated (See Table 3). Several studies have found these as risk factors for depression in the elderly [36,46,47]. A study done on adult females in Pakistan also concurred with our finding [41]. The finding that the elderly who had lost their spouse were suffering from a higher rate of depression could be explained by the fact that late life support by the partner is of importance to their psychological health. Dependence of the elderly on their spouse increases as they age. Death of a spouse renders them vulnerable to mental stress. Indeed, widowhood has been found to be strongly associated with depression in several instances [48,49].

Circumstances which lead to divorce or separation, especially if it occurs at a late stage, can lead to adjustment problems, which may manifest as depressive symptoms. A study showed that marital disruption was associated with a higher prevalence of major depression in both men and women [50]. In addition, people who remain single lack children and spousal support. Life-events hence are much more unbearable, especially at an old age. Such factors may inevitably lead to psychological stress and depression.

### Employment Status

As populations stop working, they lose not only the economic but also the social and psychological benefits of activity and purpose [51]. Ageing and employment, ideally have an inverse relationship, with global statistics indicating a mean retirement from employment at the age of 61–62 years [52]. This is not entirely representative of a developing nation such as Pakistan, where low socio-economic resources per household compel the aged to continue working. Although the formal retirement age in Pakistan is 60, individual companies allot retirement ages as per requirement. Even for an urban setting, limited or no retirement benefits (gratuity, provident funds) press the elderly for seeking yet another job, non-availability of which renders them stressed and under financial burden. Such a scenario serves as a nidus for depression at this age, which is supported by our finding that unemployed/retired subjects were twice more likely to suffer from depression than those who were employed. While there are health benefits of an early retirement in some parts of the world, these benefits do not always adequately cover the increasing medical costs that accompany age [51]. The association of unemployment and depressive symptoms has been extensively studied. Studies have revealed that unemployment can give rise to reduced hope and financial problem, which in turn contribute to depression. [53-55].

### Other socio-demographic variables

Other socio-demographic variables which achieved significance on univariate analysis but not on multivariate analysis included: living alone and being childless. The elderly who lived alone were found to be at a high risk of depression [56], which was consistent with our findings. One study showed that being childless had no direct role in predisposing the elderly to depression [57]. However our study was not in accordance.

The absence of a caregiver was deduced to be a possible risk factor for depression. However, we did not find any significant association with depression in our study. One possible reason for this finding could be that we did not ask the number of caregivers or who the caregiver was. The elderly who were residing in a joint family system were likely to have more caregivers as well as more adult children as caregivers.

### Limitations

The main limitation of our study was the use of a translated version of the GDS-15. This was done due to the unavailability of a validated Urdu version of the GDS-15. Although it is possible that the GDS-15 may not retain its high psychometric values after our translation, we believe that the values will not differ greatly. The wordings and meaning of the questions were preserved after back translation to English. We used a cut-off value of 5 to identify depression. This value has been shown to be highly sensitive and specific in other populations [26,27]. However, the cut-off for depression may differ in different populations. This may introduce bias in our results.

Studies have shown that the psychometric properties of the GDS are weaker when used on people with cognitive impairment [23]. Screening for people with cognitive impairment was not done in our study.

Since we collected the sample from only one location in Karachi, the generalizability of our results may be restricted. The generalizabilty is also affected by the fact that since this was a hospital survey, a fair proportion of the sample consisted of patients. It is known that people with co-morbids are more likely to suffer from depression [32,40]; therefore, the prevalence of depression in our sample may be over-estimated. However, due to the central location of the hospital, it is still a good site for collection of this sample, as it receives people from all over the city.

## Conclusion

The present study found that residing in a nuclear family system is a strong independent predictor of depression in the elderly. The prevalence of depression in the elderly population in our study was moderately high and a cause of concern. Since the hospital draws people from all over Karachi, it can be postulated that the prevalence of depression in the elderly in Karachi is about one in five.

The progressive urbanization in a city like Karachi is inevitable. The nucleation of family systems is likely to follow. This transition in family systems may have a major deleterious effect on the physical and mental health of the elderly. Social support programs for the elderly especially females must be developed to ensure their well-being.

## List of abbreviations used

Joint Family System (JFS), Nuclear Family System (NFS), Geriatric Depression Scale (GDS), Aga Khan University and Hospital (AKUH), Community Health Centre (CHC).

## Competing interests

The authors declare that they have no competing interests.

## Authors' contributions

WQ was involved in the conception of the study and its ongoing management. AMT and AI designed the study and were involved in the data collection, data analysis, data interpretation and preparation of the manuscript. ZQ contributed to the data analysis and data interpretation. All authors read and approved the final manuscript.

## Pre-publication history

The pre-publication history for this paper can be accessed here:



## Supplementary Material

Additional file 1 English version of questionnaire. This is the English version of the questionnaire. Click here for file

Additional file 2 Urdu version of questionnaire – Page 1. This is page 1 of Urdu version of the questionnaire. Click here for file

Additional file 3 Urdu version of questionnaire – Page 2. This is page 2 of Urdu version of the questionnaire. Click here for file
