# Supplementary material for: Depression in the elderly: Does family system play a role? A cross-sectional study
Source: BMC Psychiatry. 2007 Oct 25;7:57. doi: 10.1186/1471-244X-7-57 (PMC2194680; doi:10.1186/1471-244X-7-57)
Supplement: Additional file 1 — English version of questionnaire. This is the English version of the questionnaire. [file 1471-244X-7-57-S1.doc]

**Depression in The Elderly: Does Family System Play a Role?**

**1.1** Age ⁪⁪⁪

**1.2** Sex 1.Male ⁪ 2.Female ⁪

**1.3** Marital Status:

1.Single ⁪ 2.Married ⁪ 3.Divorced ⁪ 4.Widowed/widower ⁪ 5.Separated ⁪

**1.4** Education:

1.Illiterate ⁪ 2.Can read/write ⁪ 3.Primary ⁪ 4.Secondary ⁪ 5.Intermediate ⁪ 6.Graduate ⁪ 7.Postgraduate ⁪ 8.Diploma ⁪

**1.5** Religion:

1.Islam ⁪ 2.Christianity ⁪ 3.Hindu ⁪

4.Other (Please Specify) ____________________

**1.6** Employment Status:

1. Employed ⁪ 2.Unemployed ⁪ 3. Student ⁪

4. Housewife ⁪ 5. Self employed ⁪

6. Retired ⁪

7. Other (Please Specify)­­­­­­­­­­­­­­______________________

**1.7** You are living (more than one could be checked):

1.Alone ⁪ 2.With Spouse ⁪ 3.With Children ⁪ 4.Other (Please Specify) __________________

**1.8** If you have children but they are not living with you, they are living:

1. In the same city as you ⁪ 2. In the same country as you ⁪ 3. Abroad ⁪

**1.9** If you have children but they are not living with you, how often do they visit?

1. Every day ⁪ 2.1-2 times per week ⁪

3. Once per month ⁪ 4. Less than once per month ⁪

**1.10** Who is your primary care giver?

1.None ⁪ 2.Spouse ⁪ 3.Child ⁪

4. Private Nurse ⁪ 5. Servant ⁪ 6.Other (please specify) ________

**1.11** Your financial support is derived by/from:

1.Self ⁪ 2.Children ⁪ 3.Pension-private ⁪

4.Pension-government ⁪ 5.Charity ⁪ 6.Others (please specify) __________

**1.12** What kind of a family system are you presently living in? (The meaning of each family system is explained here)

1. Joint Family System ⁪ 2. Nuclear Family System ⁪

**GERIATRIC DEPRESSION SCALE (GDS – 15)**

| **2.0** - Question | Yes No |
| --- | --- |
| 1. Are you basically satisfied with your life? | ⁪ ⁪ |
| 2. Have you dropped many of your activities and interests? | ⁪ ⁪ |
| 3. Do you feel happy most of the time? | ⁪ ⁪ |
| 4. Do you prefer to stay at home, rather than going out and doing new things? | ⁪ ⁪ |
| 5. Are you in good spirits most of the time? | ⁪ ⁪ |
| 6. Do you think it is wonderful to be alive now? | ⁪ ⁪ |
| 7. Do you feel full of energy? | ⁪ ⁪ |
| 8. Do you feel that your life is empty? | ⁪ ⁪ |
| 9. Do you often get bored? | ⁪ ⁪ |
| 10. Are you afraid that something bad is going to happen to you? | ⁪ ⁪ |
| 11. Do you often feel helpless? | ⁪ ⁪ |
| 12. Do you feel you have more problems with memory than most? | ⁪ ⁪ |
| 13. Do you feel that your situation is hopeless? | ⁪ ⁪ |
| 14. Do you think that most people are better off than you are? | ⁪ ⁪ |
| 15. Do you feel pretty worthless the way you are now? | ⁪ ⁪ |

Total GDS score: ___

(1) 0 - 4 Normal (No depression) [ ]

(2) 5 - 15 Screen positive for depression [ ]
