# Supplementary figures and images for: Depression in the elderly: Does family system play a role? A cross-sectional study
Source: BMC Psychiatry. 2007 Oct 25;7:57. doi: 10.1186/1471-244X-7-57 (PMC2194680; doi:10.1186/1471-244X-7-57)

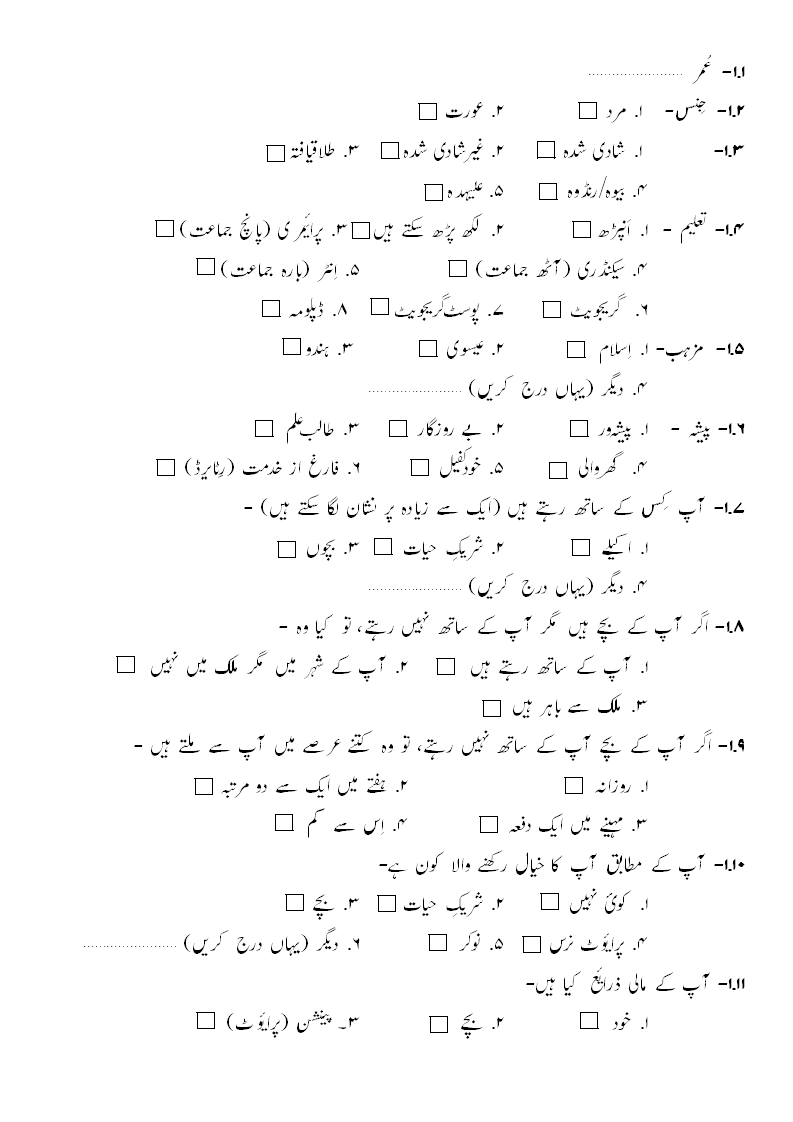

Supplement: Additional file 2 — Urdu version of questionnaire – Page 1. This is page 1 of Urdu version of the questionnaire. [file 1471-244X-7-57-S2.gif]

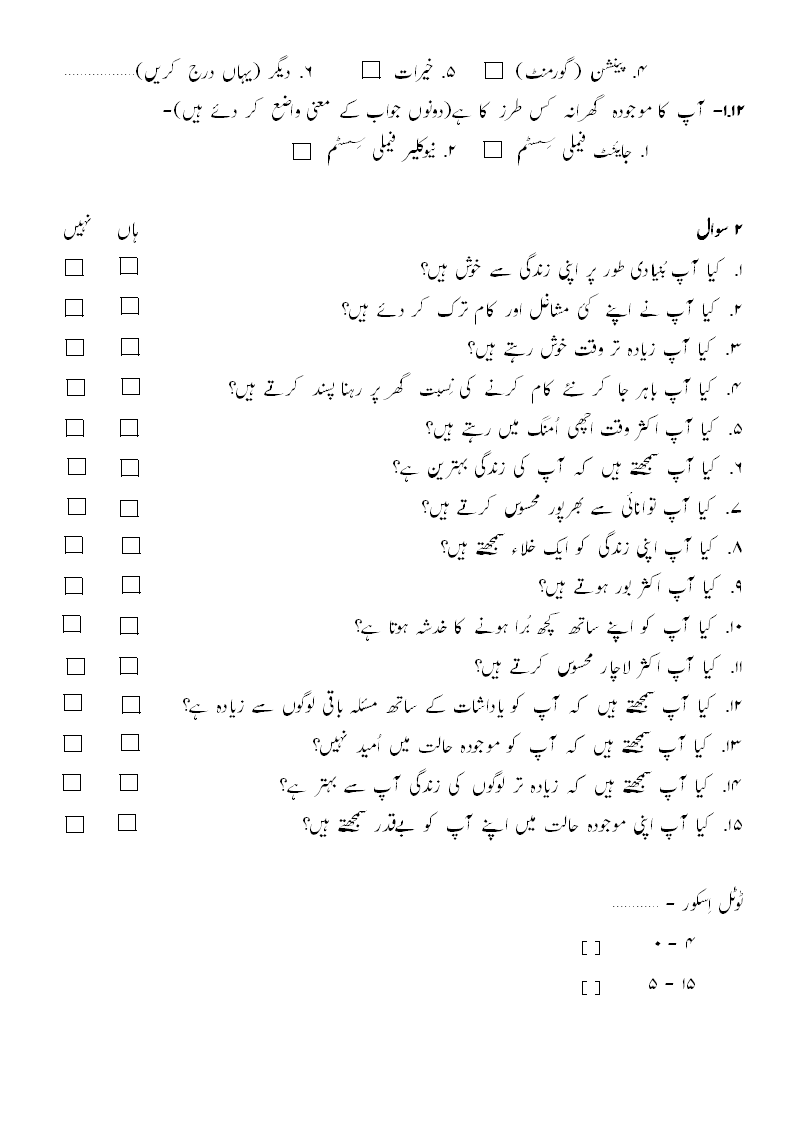

Supplement: Additional file 3 — Urdu version of questionnaire – Page 2. This is page 2 of Urdu version of the questionnaire. [file 1471-244X-7-57-S3.gif]
